# Model-based myocardial T1 mapping with sparsity constraints using single-shot inversion-recovery radial FLASH cardiovascular magnetic resonance

**DOI:** 10.1186/s12968-019-0570-3

**Published:** 2019-09-19

**Authors:** Xiaoqing Wang, Florian Kohler, Christina Unterberg-Buchwald, Joachim Lotz, Jens Frahm, Martin Uecker

**Affiliations:** 1 Department of Diagnostic and Interventional Radiology, University Medical Center Göttingen, Robert-Koch-Str. 40, 37075 Göttingen, Germany; 2 DZHK (German Centre for Cardiovascular Research), partner site Göttingen, Berlin, Germany; 3 Biomedizinische NMR, Max-Planck-Institut für biophysikalische Chemie, Am Fassberg 11, 37077 Göttingen, Germany

**Keywords:** Model-based reconstruction, Myocardial T1 mapping, Sparsity constraint, Radial FLASH

## Abstract

**Background:**

This study develops a model-based myocardial T1 mapping technique with sparsity constraints which employs a single-shot inversion-recovery (IR) radial fast low angle shot (FLASH) cardiovascular magnetic resonance (CMR) acquisition. The method should offer high resolution, accuracy, precision and reproducibility.

**Methods:**

The proposed reconstruction estimates myocardial parameter maps directly from undersampled k-space which is continuously measured by IR radial FLASH with a 4 s breathhold and retrospectively sorted based on a cardiac trigger signal. Joint sparsity constraints are imposed on the parameter maps to further improve T1 precision. Validations involved studies of an experimental phantom and 8 healthy adult subjects.

**Results:**

In comparison to an IR spin-echo reference method, phantom experiments with T1 values ranging from 300 to 1500 ms revealed good accuracy and precision at simulated heart rates between 40 and 100 bpm. In vivo T1 maps achieved better precision and qualitatively better preservation of image features for the proposed method than a real-time CMR approach followed by pixelwise fitting. Apart from good inter-observer reproducibility (0.6% of the mean), in vivo results confirmed good intra-subject reproducibility (1.05% of the mean for intra-scan and 1.17, 1.51% of the means for the two inter-scans, respectively) of the proposed method.

**Conclusion:**

Model-based reconstructions with sparsity constraints allow for single-shot myocardial T1 maps with high spatial resolution, accuracy, precision and reproducibility within a 4 s breathhold. Clinical trials are warranted.

**Electronic supplementary material:**

The online version of this article (10.1186/s12968-019-0570-3) contains supplementary material, which is available to authorized users.

## Background

Quantitative myocardial T1 mapping finds increasing applications in clinical cardiovascular magnetic resonance (CMR) imaging. For example, native myocardial T1 mapping can be used to detect myocardial edema, while T1 maps after contrast agent are helpful for the detection of fibrosis and/or storage diseases [1, 2]. To date, developments have enabled fast cardiac T1 mapping in a clinically acceptable time, i.e., from 11 to 17 heartbeats within one breathhold. Representative techniques include modified Look-Locker inversion recovery (MOLLI) [3], short modified Look-Locker inversion recovery (shMOLLI) [4], saturation recovery single-shot acquisition (SASHA) [5], and saturation pulse prepared heart rate independent inversion recovery (SAPPHIRE) [6]. Although MOLLI and variants are the most widely used techniques [2], they still face several challenges: (1) the occurrence of banding artifacts, in particular at high field strengths, which are due to balanced steady state free precession (bSSFP) off-resonance effects, (2) the underestimation of T1 values due to an imperfect physical modeling, and (3) a breathhold time of 11 to 17 heartbeats which may be challenging for patients. Several ideas have been proposed to overcome these limitations. For example, replacing the bSSFP readout by a fast low angle shot (FLASH) acquisition completely avoids banding artifacts [7–11]. More complex physical models, which take care of the inversion efficiency or slice profile effects improve the accuracy of T1 estimation [8, 12]. More recently, non-Cartesian acquisition schemes (mainly radial) have been employed to enable fast myocardial T1 mapping [9–11]. Specifically, the combination of radial encoding with sliding window image reconstruction [10], compressed sensing [9] and real-time CMR [11] has enabled high-resolution myocardial T1 mapping within a single inversion-recovery (IR) relaxation process.

Model-based reconstructions [13–21] represent another strategy to accelerate quantitative parameter mapping in general. Such methods exploit inherent data redundancy by estimating parameter maps directly from an undersampled k-space for a known signal model [14]. With respect to T1 mapping, it has been proposed to iteratively optimize model parameters by alternating between k-space and image-space [17] with applications to the brain and heart [22]. On the other hand, recent developments formulate T1 estimation as a nonlinear inverse problem [19–21, 23]. In this way, a priori information such as sparsity constraints can be easily incorporated into the reconstruction to increase performance and in particular improve T1 accuracy and precision.

In this work, we extend a previously developed method [20] for sparsity-constrained model-based T1 estimation to allow for cardiac applications. The data acquisition is based on a single-shot IR radial FLASH sequence and triggered to early diastole. The proposed method is validated for an experimental phantom at simulated heart rates and in vivo studies with 8 healthy subjects.

## Methods

### Data acquisition and model-based reconstruction

The single-shot IR scheme used here has been reported before [11]. For myocardial T1 mapping, data acquisition starts with a non-selective inversion pulse which is triggered to the early diastolic phase with use of a finger pulse signal. After inversion, the signal is continuously acquired for a period of 4 s using a radial FLASH readout with a golden-angle trajectory. To eliminate motion effects during systolic contraction and expansion, only data from the diastolic phase is retrospectively selected for T1 mapping.

The signal from multiple coils is given by

1 $$ {\mathrm{y}}_j(t)=\int {M}_{t_k}\left(\overrightarrow{r}\right){c}_j\left(\overrightarrow{r}\right){e}^{-i\overrightarrow{r}\overrightarrow{k}(t)}d\overrightarrow{r} $$

with *c*_*j*_ the jth coil sensitivity map, $\overrightarrow{k}(t)$ the chosen k-space trajectory, y_*j*_(*t*) the acquired data and ${M}_{t_k}\left(\overrightarrow{r}\right)$ the magnetization at time *t*_*k*_ after inversion

2 $$ {M}_{t_k}={M}_{ss}-\left({M}_{ss}+{M}_0\right)\cdotp {e}^{-{t}_k\cdotp {R}_1^{\ast }} $$

where *t*_*k*_ is defined as center of the acquisition window in this study. ${M}_{ss},{M}_0\ \mathrm{and}\kern0.5em {R}_1^{\ast }$ represent the steady-state signal, equilibrium signal and effective relaxation rate, respectively. After estimation of $\left({M}_{ss},{M}_0,{R}_1^{\ast}\right)$, T1 can be calculated by

3 $$ \mathrm{T}1=\frac{M_0}{M_{ss}\cdot {R}_1^{\ast }}\kern0.5em $$

In Eqs. (1) and (2), both the model parameters ${\left({M}_{ss},{M}_0,{R}_1^{\ast}\right)}^T\ \mathrm{and}\ \mathrm{all}\ \mathrm{coil}\ \mathrm{sensitivity}\ \mathrm{maps}\ {\left({c}_1,\cdots, {c}_N\right)}^T$ are unknowns, which are directly estimated from k-space using a sparsity constrained model-based reconstruction, i.e.,

4 $$ \hat{x}=\mathrm{argmin}{\left\Vert F(x)-y\right\Vert}_2^2+\alpha R\left({x}_{\boldsymbol{p}}\right)+\beta Q\left({x}_{\boldsymbol{c}}\right) $$

Here *F* is the nonlinear forward model mapping all unknowns to the measured data *y*:

5 $$ F:x\mapsto \left(\begin{array}{c}{P}_1\mathcal{F}\left\{{c}_1\cdot {M}_{t_1}\left({M}_{ss},{M}_0,{R}_1^{\ast}\right)\right\}\\ {}\vdots \\ {}{P}_1\mathcal{F}\left\{{c}_N\cdot {M}_{t_1}\left({M}_{ss},{M}_0,{R}_1^{\ast}\right)\right\}\\ {}{P}_2\mathcal{F}\left\{{c}_1\cdot {M}_{t_2}\left({M}_{ss},{M}_0,{R}_1^{\ast}\right)\right\}\\ {}\vdots \\ {}{P}_n\mathcal{F}\left\{{c}_N\cdot {M}_{t_n}\left({M}_{ss},{M}_0,{R}_1^{\ast}\right)\right\}\end{array}\right) $$

with *P* the orthogonal projection onto the trajectory and $\mathcal{F}$ the 2D Fourier transform. The unknowns ${x}_{\boldsymbol{p}}={\left({M}_{ss},{M}_0,{R}_1^{\ast}\right)}^T$ and *x*_***c***_ = (*c*_1_, ⋯, *c*_*N*_)^*T*^. *R*(*x*_***p***_) is a L1-Wavelet regularization which exploits joint sparsity in the parameter dimension following the ideas of compressed sensing, while *Q*(*x*_***c***_) is a Sobolev norm which is applied to the coil sensitivities to enforce their intrinsic smoothness. α and β are the corresponding regularization parameters. The nonlinear inverse problem in Eq. (4) is solved by the iteratively regularized Gauss-Newton method (IRGNM) [24] where the nonlinear problem is linearized in each Gauss-Newton step and solved by the fast iterative shrinkage-thresholding algorithm (FISTA) [25]. More details of the IRGNM-FISTA algorithm can be found in [20].

### CMR

All CMR studies were conducted on a 3 T system (Magnetom Skyra, Siemens Healthineers, Erlangen, Germany) with approval of the local ethics committee. Phantom measurements employed a 20-channel head/neck coil, while human heart studies used a combined thorax and spine coil with 26 channels. Eight subjects (three female, five male, age 27 ± 3, range 23–32 years; heart rates 62 ± 11 bpm, range 50–80 bpm) with no known illness were recruited. Written informed consent was obtained from all subjects prior to CMR. In vivo T1 measurements were performed within a single breathhold.

The proposed method was experimentally validated at simulated heart rates with a commercial reference phantom (Diagnostic Sonar LTD, Livingston, Scotland, UK) consisting of six compartments with defined T1 values surrounded by water. The gold standard T1 map for the phantom was estimated using an IR spin-echo method [26] with 9 IR scans (TI = 30, 530, 1030, 1530, 2030, 2530, 3030, 3530, 4030 ms), TR/TE = 4050/12 ms, FOV 192 × 192 mm^2^, matrix size 192 × 192, and a total acquisition time of 2.4 h.

For IR radial FLASH, continuous data acquisition was performed with a tiny golden angle (18.71°) [27] after non-selective inversion. Because there is no intermediate image reconstruction, model-based reconstructions offer a flexible choice of temporal resolution, i.e., they allow a combination of an arbitrary (small) number of radial spokes for each k-space frame. However, as long as the T1 accuracy is not compromised, a certain degree of temporal discretization (data binning) is recommended to reduce the computational demand [19, 20]. In this study, 17 spokes formed one k-space and resulted in a temporal resolution of 45 ms. According to the subjects’ heart rates, the resulting number of k-space frames were 48 ± 9, range 33–57 for reconstructions in this study. Single-shot myocardial T1 maps of the mid-ventricular slices were acquired at a nominal in-plane resolution of 1.0 × 1.0 mm^2^ and 8 mm slice thickness using a FOV 256 × 256 mm^2^ in combination with a resolution of 512 complex data points per radial spoke (two-fold oversampling). Other parameters were TR/TE = 2.67/1.67 ms, nominal flip angle 6°, bandwidth 850 Hz/pixel and total acquisition time 4 s.

To access reproducibility of the proposed method, the single-shot sequence was performed 3 times on each subject: The first two measurements were repeated one after the other, while the third one was done with a 5-min break, during which time the subject was taken out of the scanner. For comparisons, single-shot T1 maps were also estimated using the frame-based nonlinear inversion (NLINV) reconstruction with subsequent pixel-wise fitting as described in [11] without and with spatial filtering by a modified nonlocal means filter [28] from the same datasets. Further, a 5(3)3 MOLLI sequence provided by the vendor was applied for reference using a FOV of 360 × 306.6 mm^2^, in-plane resolution 1.41 × 1.41 × 8 mm^3^, TR/TE = 2.24/1.12 ms, nominal flip angle 35°, bandwidth 1085 Hz/pixel and total acquisition time 11 heart beats.

### Implementation

All data was processed off-line. Multicoil raw data were first corrected for gradient delays [29] and then compressed to 10 virtual channels using a principal component analysis (PCA). A convolution-based gridding [30] without density compensation was used to interpolate the radial samples onto a Cartesian grid on which all successive iterations were performed. All the computations were done in Berkeley advanced reconstruction toolbox (BART) [31] on a 40-core 2.3 GHz Intel Xeon E5–2650 PC with a RAM size of 500 GB.

The parameter maps ${\left({M}_{ss},{M}_0,{R}_1^{\ast}\right)}^T\ \mathrm{were}\ \mathrm{initialized}\ \mathrm{with}\ {\left(1.0,1.0,1.5\right)}^T$ and all coil sensitivities zeros for all reconstructions. 10 Gauss-Newton steps were employed to ensure convergence. Similar to [20], regularization parameters α and β were initially set to 1 and subsequently reduced by a factor of 3 in each Gauss–Newton step. A minimum value of α was used to control the noise at higher Gauss–Newton steps. The chosen value of *α*_min_ was defined by optimizing signal to noise ratio (SNR) without compromising quantitative accuracy or delineation of structural details. With the above settings, the whole computation took around 6 h using the CPUs. However, with a reduced number (e.g., 6) of virtual coils, computations could be run on a GPU, which took 10 to 20 min per dataset

### Data analysis

Results in this work are reported as mean ± standard deviation (SD). For the assessment of myocardial T1 values, the regions of interest (ROIs) in the inter-ventricular septum were carefully selected to exclude the blood pool using arrShow [32] tool in MATLAB (MathWorks, Natick, Massachusetts, USA) and performed by two independent observers. Similar to [8, 33], the precision of T1 estimation was evaluated using coefficient of variation (CV = SD_ROI_/Mean_ROI_ × 100%). The reproducibility error was calculated by $\sqrt{\left({\sum}_{i=1}^{n_s}\mathrm{T}{1}_{\mathrm{diff}}^2(i)\right)/{n}_s},$ where T1_diff_(*i*) is the T1 difference between different measurements, *n*_s_ is the number of subjects. Further, a repeated measures analysis of variance (ANOVA) with Bonferroni post hoc test was used for comparisons and a *P* value < 0.05 was considered significant.

In addition, edge sharpness was quantitatively measured for both the proposed model-based reconstruction and MOLLI. It was done by fitting each septal T1 line profile (starting from the blood pool to the middle of the myocardial septum) to a parameterized sigmoid function [34]: $s\left(\mathrm{x}\right)=\frac{\mathrm{a}}{1+{\mathrm{e}}^{-\mathrm{k}\cdot \left(\mathrm{b}-\mathrm{x}\right)}}+c$, where x is the length (unit: millimeter) along the line profile and (a, b, c, k)^*T*^ are the fitting parameters: a determines the vertical range, b determines the center location, c defines the vertical offset and k quantifies the growth rate or sharpness of the edges (The higher |k|, the sharper the edges). The above nonlinear least square fitting was then performed in MATLAB (MathWorks) using the Levenberg-Marquardt algorithm with a stopping criteria similar to [11].

## Results

**Fig. 1:**
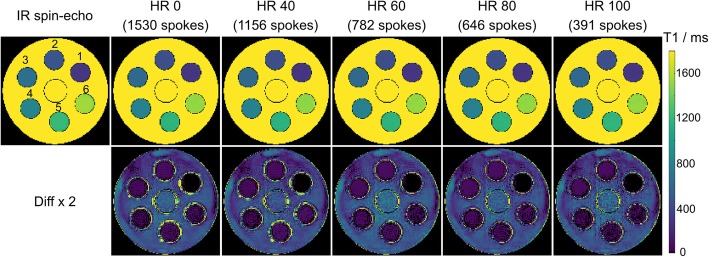
(Top) Model-based T1 maps and (bottom) T1 difference maps (× 2) for an experimental phantom and simulated heart rates (HR) in comparison to an inversion recovery (IR) spin-echo reference method

Figure 1 shows estimated T1 maps of an experimental phantom for different simulated heart rates between 40 and 100 bpm. The proposed technique is compared to a reference T1 map obtained by a conventional IR spin-echo method. Zero heart rate refers to a situation where no k-space data is deleted prior to model-based reconstruction. Visual inspection reveals good agreement for all heart rates and T1 values. These qualitative findings are confirmed by quantitative analyses summarized in Table 1. The maximum deviation between the proposed method and the reference is 10%. Noteworthy, good precision is preserved at high heart rates for the proposed method. A long-axis T1 mapping was further performed (Additional file 1: Figure S1) to validate robustness of the proposed method. Both visual inspection and quantitative results (Additional file 3: Table S1) confirmed good T1 accuracy and precision in the long-axis view as well.

**Table 1:** T1 relaxation times (ms) for an experimental phantom and simulated heart rates

Tube	IR spin-echo	T2 [35]	HR 0	HR 40	HR 60	HR 80	HR 100
1	315 ± 6	101 ± 2	308 ± 5	309 ± 5	308 ± 5	307 ± 5	310 ± 6
2	497 ± 8	46 ± 2	452 ± 5	453 ± 5	449 ± 5	448 ± 5	452 ± 7
3	661 ± 8	81 ± 3	622 ± 6	622 ± 5	618 ± 5	615 ± 5	617 ± 7
4	822 ± 12	132 ± 5	793 ± 7	792 ± 9	786 ± 8	787 ± 9	793 ± 10
5	1191 ± 13	138 ± 4	1149 ± 15	1140 ± 15	1148 ± 13	1153 ± 15	1159 ± 17
6	1508 ± 15	166 ± 5	1474 ± 14	1462 ± 16	1479 ± 17	1474 ± 17	1480 ± 21

*HR* Heart rate, *IR* Inversion recovery

**Fig. 2:**
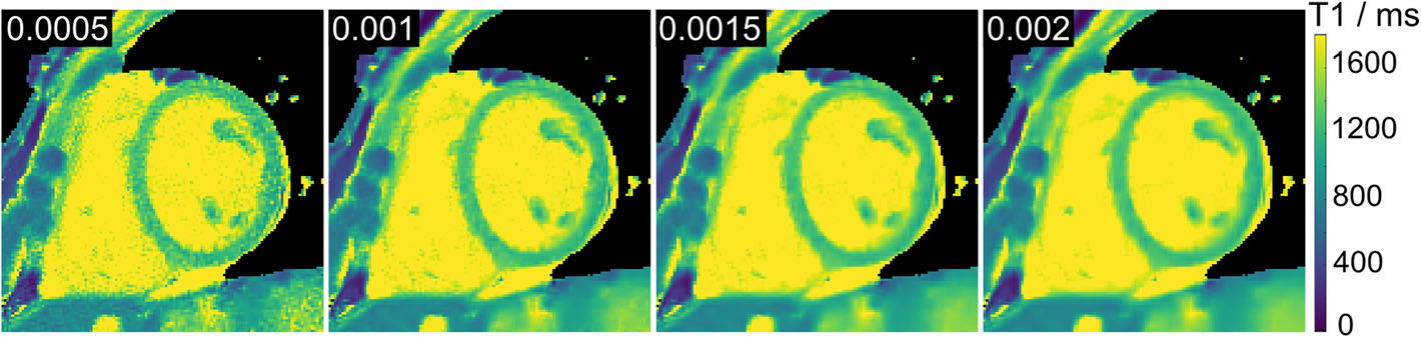
Model-based myocardial native T1 maps as a function of the minimum regularization parameter *α*_min_. A value *α*_min_ = 0.0015 is used for all in vivo studies

**Fig. 3:**
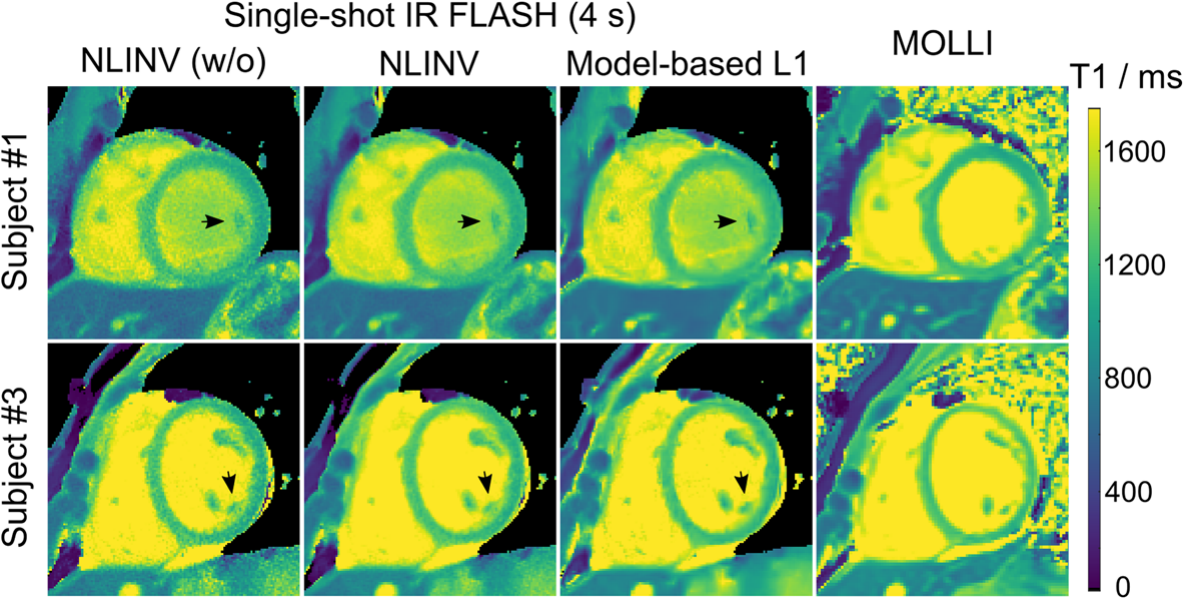
Myocardial T1 maps obtained by single-shot IR radial fast low angle shot (FLASH) using (leftmost column) nonlinear inversion (NLINV) without spatial denoising and (middle left column) NLINV with spatial denoising and (middle right column) sparsity-constrained model-based reconstruction versus MOLLI. Black arrows indicate better preservation of image features for the proposed method

Figure 2 demonstrates the influence of the minimum regularization parameter *α*_min_ used in sparsity − regularized model − based reconstructions. Low values of *α*_min_ increase noise in the myocardial T1 maps, while high values lead to blurring. A value of *α*_min_ = 0.0015 was chosen to balance between noise reduction and preservation of image details. With these settings, Fig. 3 compares myocardial T1 maps of two representative subjects obtained by the proposed model-based reconstruction versus a MOLLI technique and NLINV approaches without and with spatial filtering. In comparison to the NLINV approaches, model-based reconstructions generate T1 maps with visually less noise and better qualitative preservation of image features as indicated by black arrows. Table 2 shows quantitative T1 data for the left-ventricular septum of all subjects. The repeated measures ANOVA tests of the quantitative results revealed no significant difference among the quantitative mean myocardial T1 values by NLINV approaches and model-based reconstructions: NLINV (w/o) versus NLINV versus model-based: 1239 ± 16 versus 1244 ± 16 versus 1243 ± 15 ms (*p* = 0.37). However, the CV values are significantly different: NLINV (w/o) versus NLINV versus model-based: 5.7% ± 0.7% versus 3.1% ± 0.2% versus 3.1% ± 0.2% (*p* < 0.01). A post hoc Bonferroni test confirmed that both the proposed model-based reconstruction and NLINV with the denoising filter have lower CV values, i.e., better T1 estimation precision than the NLINV method without spatial filtering (*p* < 0.01).

**Table 2:** Myocardial T1 values (ms) and CVs in left-ventricular septum of eight subjects using single-shot IR fast low angle shot (FLASH) with nonlinear inversion (NLINV) reconstruction without and with a spatial filter, the proposed model-based reconstruction and modified Look-Locker inversion recovery (MOLLI), respectively

Subject	Age/years	HR/bpm	NLINV(w/o)		NLINV		Model-based L1		MOLLI	
			T1 (ms)	CV	T1 (ms)	CV	T1 (ms)	CV	T1 (ms)	CV
1	32	55	1224 ± 55	4.5%	1232 ± 36	2.9%	1219 ± 35	2.9%	1215 ± 34	2.8%
2	24	55	1237 ± 63	5.1%	1249 ± 36	2.9%	1255 ± 36	2.9%	1212 ± 20	1.7%
3	23	50	1253 ± 61	4.9%	1268 ± 40	3.2%	1251 ± 41	3.3%	1242 ± 36	2.9%
4	30	58	1243 ± 82	6.6%	1248 ± 41	3.3%	1255 ± 38	3.0%	1226 ± 27	2.2%
5	25	60	1220 ± 73	6.0%	1224 ± 36	2.9%	1233 ± 36	2.9%	1189 ± 23	1.9%
6	27	55	1227 ± 73	5.9%	1242 ± 44	3.5%	1231 ± 39	3.2%	1195 ± 34	2.8%
7	25	80	1270 ± 82	6.5%	1268 ± 41	3.2%	1264 ± 42	3.3%	1245 ± 36	2.9%
8	27	80	1234 ± 88	6.1%	1224 ± 42	3.0%	1232 ± 45	3.2%	1210 ± 23	1.9%

**Fig. 4:**
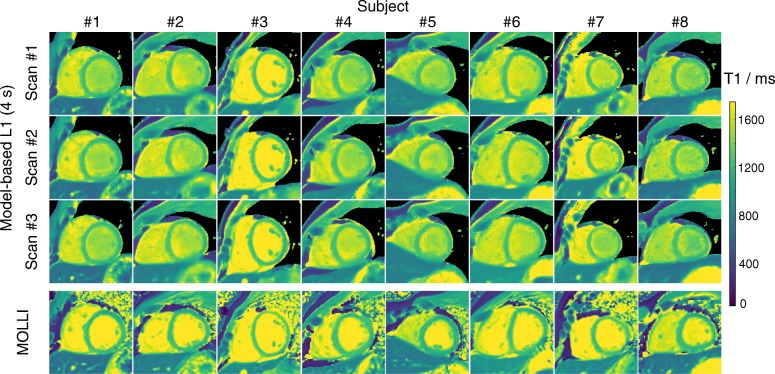
Three repetitive model-based T1 maps in comparison to MOLLI T1 maps for all 8 subjects

**Fig. 5:**
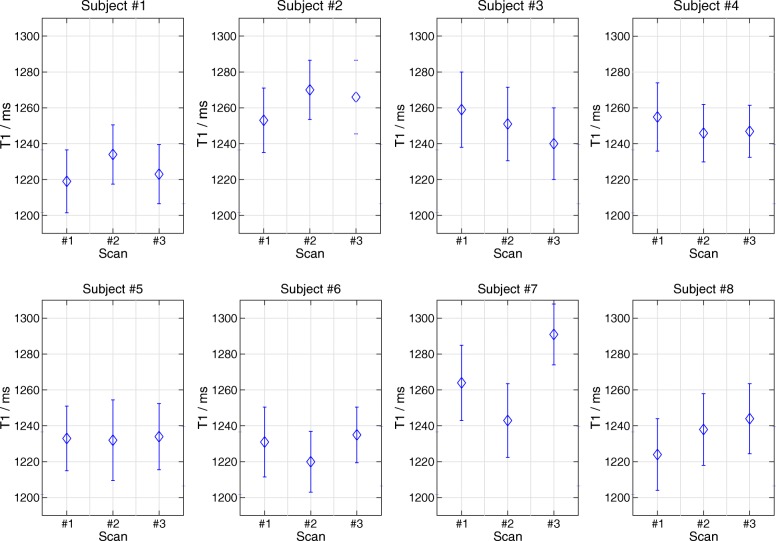
Myocardial T1 values (ms) in the mid-ventricular septal segment for all 8 subjects and three repetitive scans

Figure 4 depicts a MOLLI T1 map and three repetitive T1 maps using the proposed method for all 8 subjects. The small visual difference among the repetitive scans demonstrates good intra-subject reproducibility of the proposed method. These findings are quantitatively confirmed in Fig. 5 which presents mid ventricular septal T1 values for all subjects and all scans. The reproducibility errors for the proposed method are 14.3 ms (1.15% of the mean) for the intra-scan and 13.3 ms (1.07% of the mean), 18.8 ms (1.51% of the mean) for the two inter-scans respectively. Although slightly higher, the reproducibility errors are comparable to the corresponding values of MOLLI: 7.0 ms (0.6% of the mean), 11.7 ms (0.97% of the mean) and 13.9 ms (1.16% of the mean), respectively. Similarly, good inter-observer reproducibility was observed for both the proposed method and MOLLI, i.e., reproducibility error 7.5 ms (0.6% of the mean) and 6.4 ms (0.5% of the mean).

**Fig. 6:**
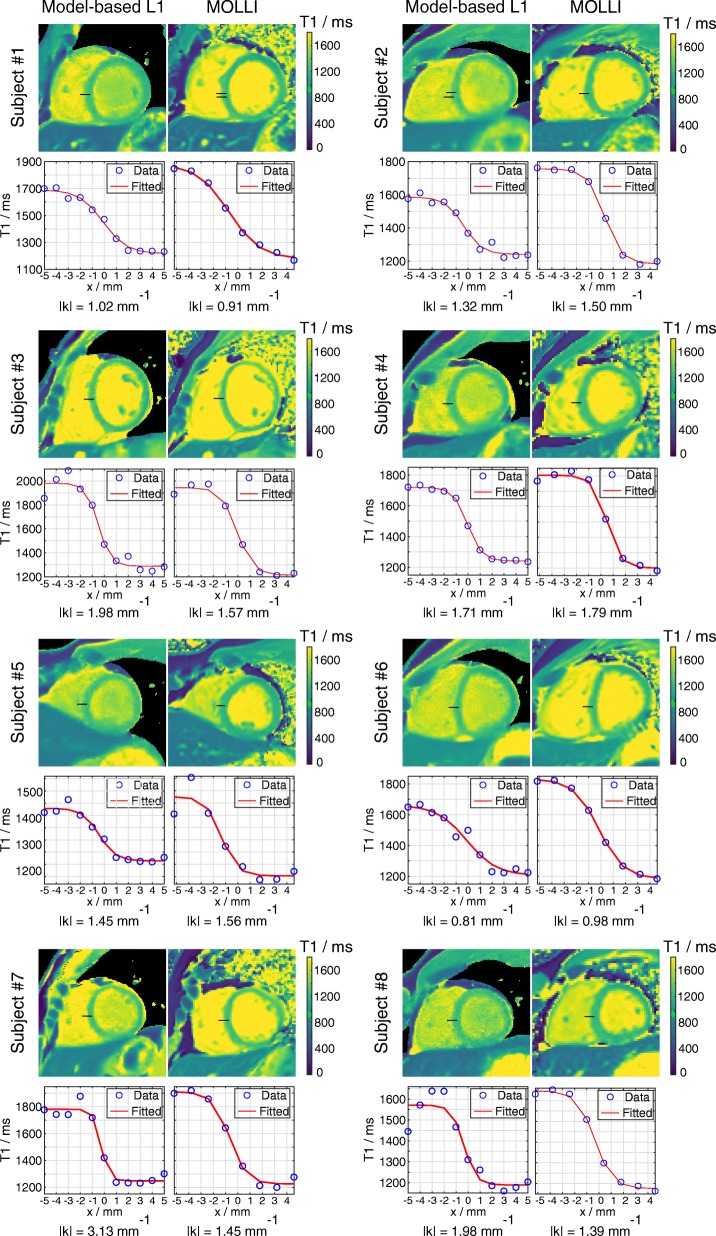
Quantitative measurements of T1 edge sharpness for the proposed method and MOLLI for 8 subjects. For each subject, (top) T1 maps estimated by the two methods, (middle) selected T1 line profiles and the fitted sigmoid curves, (bottom) the quantitative sharpness values |k|. The selected line profiles are indicated by black lines on the T1 maps

**Fig. 7:**
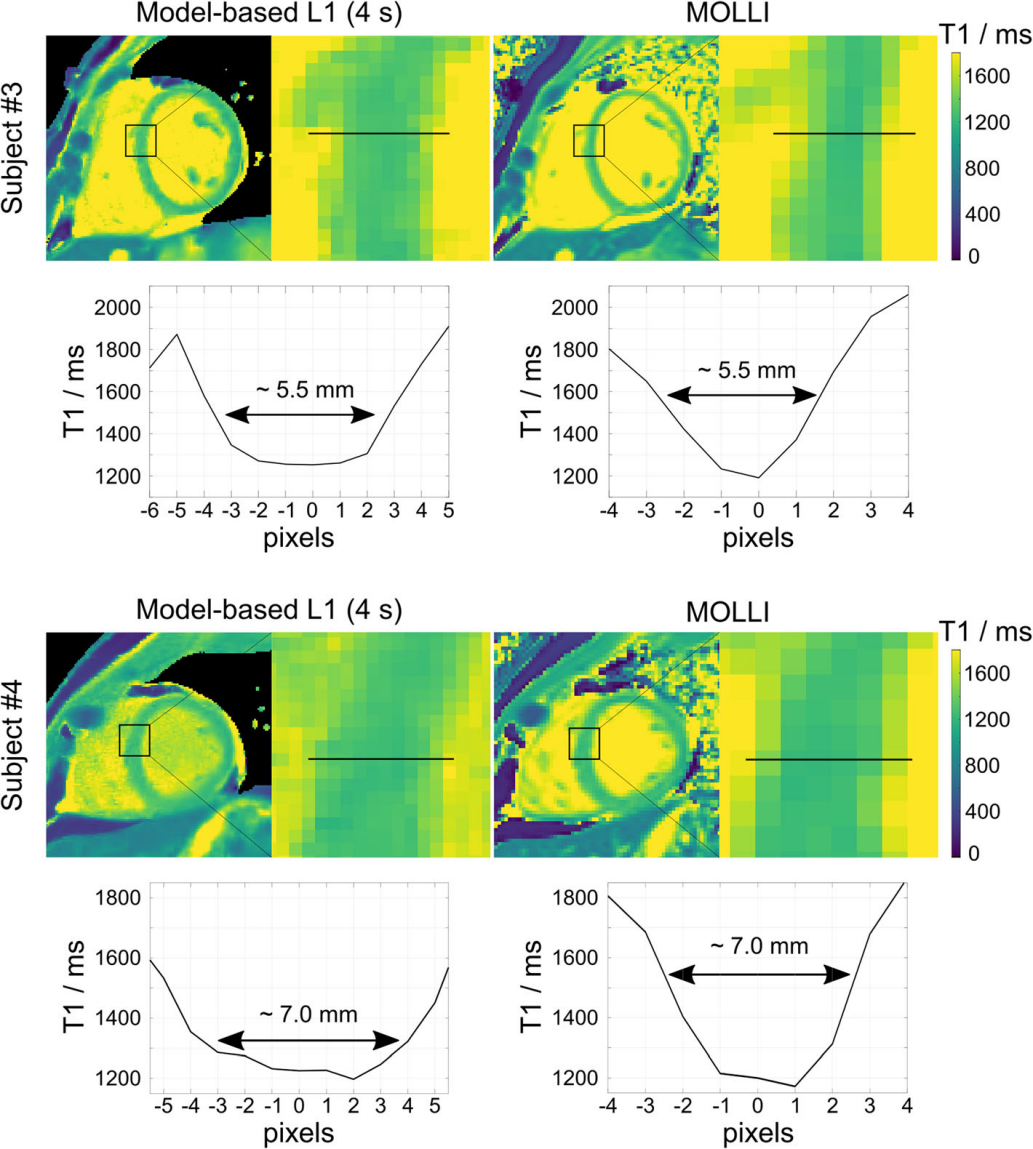
(Top) Myocardial T1 maps and (bottom) selected T1 line profiles across the mid-ventricular septum by the proposed method and MOLLI

Figure 6 shows the sharpness measurements for all T1 maps by the proposed model-based reconstruction and MOLLI. Good correspondence was observed between the selected T1 line profiles and the fitted sigmoid curves for all datasets. The quantitative sharpness values |k| presented below each T1 map revealed no significant difference between the proposed method and MOLLI (model-based versus MOLLI: 1.67 ± 0.68 versus 1.39 ± 0.28 mm^− 1^, *p* = 0.22), indicating the proposed method produces T1 maps with comparable edge sharpness to MOLLI. Figure 7 further demonstrates estimated T1 maps and selected T1 line profiles across the myocardial septum by both methods for two representative subjects. More pixels are present across the septum by the model-based reconstructions, suggesting the proposed method should be helpful in reducing partial volume errors in myocardial T1 ROI measurements.

**Fig. 8:**
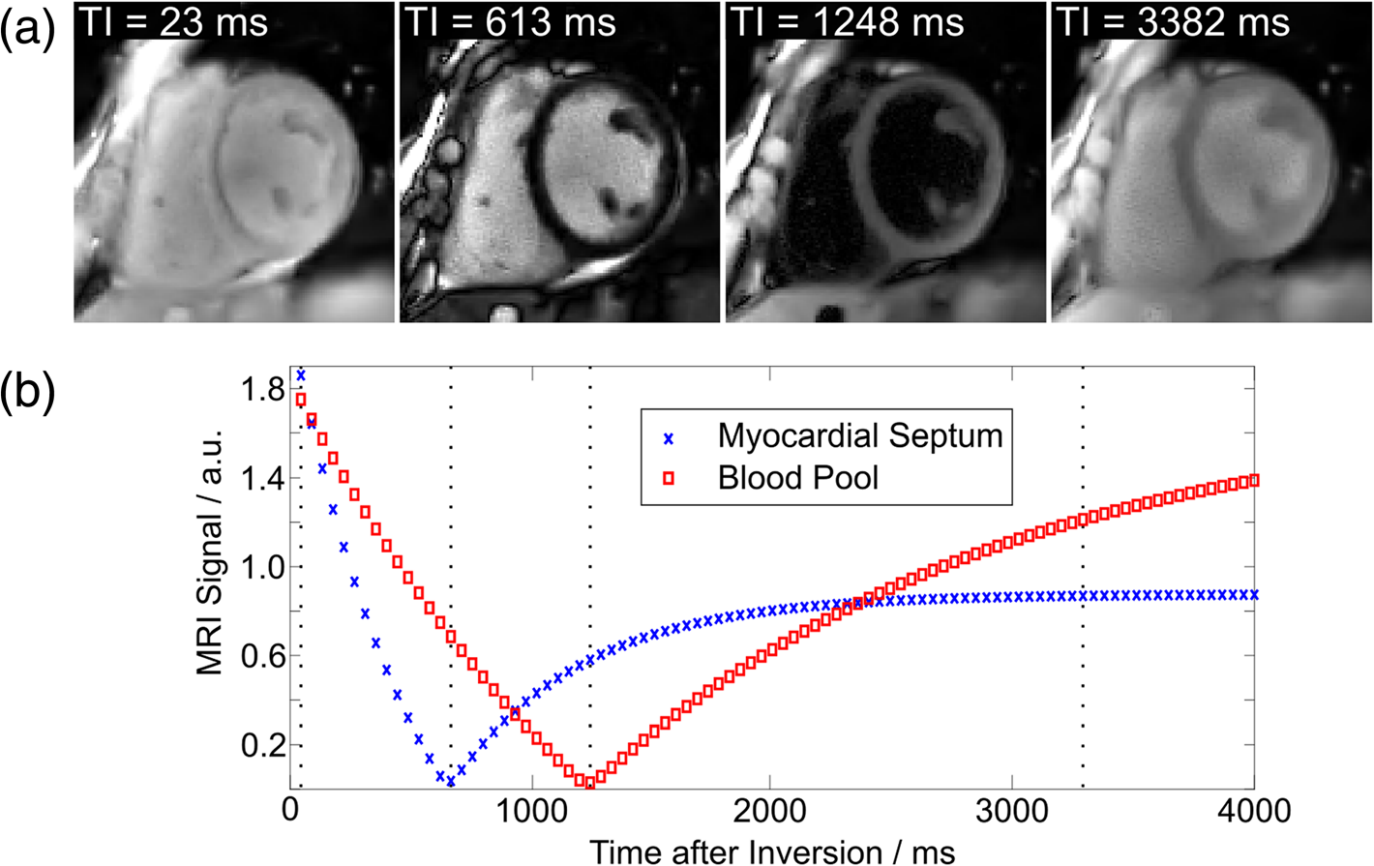
**a** Synthesized T1-weighted images at four representative inversion times. **b** Signal evolutions of myocardial septum and blood pool (ROI averaged) during inversion recovery

Apart from myocardial T1 maps, synthetic T1-weighted images can also be generated based on the signal Eq. (2) after model-based reconstructions. Figure 8a demonstrates four representative T1-weighted images starting from the beginning of inversion recovery to the time of dark blood, bright blood and steady state contrasts. The corresponding time points are also visible as dashed lines in the recovery curves in Fig. 8b. Both the dark blood and bright blood-weighted images clearly resolve contrasts between myocardium and blood pool (The whole image series with a temporal resolution of 45 ms can be found in the Additional file 4: Video S1).

## Discussion

This work presents a novel myocardial T1 mapping technique using a sparsity-constrained model-based reconstruction of a triggered single-shot IR radial FLASH acquisition. This method allows a flexible choice of temporal resolution as no intermediate image reconstruction is needed. Both studies on an experimental phantom and eight normal subjects demonstrate the proposed method could provide high-resolution myocardial T1 maps with good accuracy, precision, reproducibility and robustness within a measuring time of only 4 s. Plus, this method offers synthesized T1-weighted images with good contrast between myocardium and blood pool.

The present method is very general and not limited to the single-shot sequence employed in this work. For example, it can also be combined with a MOLLI or SASHA sequence, as both share a similar IR signal model as used here. Moreover, also a Bloch-equation based signal model [8] can be integrated into the reconstruction framework. In that case, factors such as slice profiles and inversion efficiency may be taken into consideration for an even more accurate myocardial T1 mapping. On the other hand, a further improved efficiency may be achieved by combining the current model-based reconstruction with simultaneous multi-slice (SMS) techniques [36, 37]. Such strategies will allow for simultaneous single-shot myocardial T1 mapping within multiple sections.

This study mainly focuses on diastolic T1 mapping. However, when the heart rate gets higher, less diastolic data will be available within 4 s, making the proposed method more challenging, e.g., the resulting diastolic T1 maps will get slightly noisier (Additional file 2: Figure S2). One possible solution is to increase the regularization strength. On the other hand, systolic T1 mapping could be performed instead as more systolic data will be available in that case. Such investigations will be carried out on patients with higher heart rates in our future clinical studies.

The main limitations of the proposed method are the large memory demand and the long reconstruction time which are mainly caused by the need to hold the entire multi-coil IR data in memory during iterative computation. Current implementations employ a PCA to compress the multi-coil data into several (here: 10) virtual channels to ameliorate the problem. However, the memory requirement is still high, which results in long computational time. Further optimization will include optimizing the algorithms, e.g., accelerating the linearized subproblem following the idea of T2 shuffling [38] as well as a more efficient GPU implementation.

Noteworthy, the estimated blood T1 values by the present sequence are not reliable as through-plane motion of blood flow would make the blood violate the assumed relaxation model. As a result, the present sequence may also be limited in the direct measurement of the myocardial extracellular volume (ECV). However, this might be a general problem for Look-Locker based approaches. The different blood T1 values between the proposed method and MOLLI can be attributed to the fact that the specific sequence used in the present work employed a continuous data acquisition scheme while MOLLI uses a triggered and prospective way for data acquisition.

The lack of motion estimation is another limitation for the proposed method. Although systolic data are retrospectively deleted prior to model-based reconstruction, residual nonrigid motion may still be present after sorting. This might be another reason why single-shot T1 maps by the proposed method appear slightly more blurred than motion-corrected MOLLI T1 maps provided by the vendor. Further investigation will either include a motion estimation into the model-based reconstruction or perform a motion-resolved self-gated quantitative mapping strategy similar to XD-GRASP [39] or MR multitasking [40].

## Conclusion

The proposed sparsity-constrained model-based reconstruction achieves single-shot myocardial T1 mapping within a 4 s breathhold. The method offers good accuracy, precision and reproducibility. More clinical trials are warranted.

## Additional files

Additional file 1: **Figure S1.** Model-based long-axis T1 maps at heart rates (left top) 60 and (left bottom) 100 as well as (right) the corresponding T1 line profiles for the experimental phantom study. The quantitative T1 values are in the Additional file 3: Table S1. (PNG 100 kb)

Additional file 2: **Figure S2.** Myocardial T1 maps on a healthy subject by retrospectively rejecting an increasing amount of data prior to model-based reconstructions. The amount of data deleted corresponds to heart rates 50, 60, 80, 100 bpm, respectively. The ROI-analyzed septum T1 values are 1251 ± 41 ms, 1235 ± 43 ms, 1236 ± 49 ms and 1274 ± 53 ms for each reconstruction. (PNG 149 kb)

Additional file 3: **Table S1.** Long-axis T1 relaxation times (ms) for an experimental phantom and simulated heart rates 60 and 100. (DOCX 13 kb)

Additional file 4: **Video S1.** Synthesized T1-weighted image series at a temporal resolution of 45 ms. (AVI 30000 kb)

## Data Availability

In the spirit of reproducible research, the source code of the proposed method will be made available at: https://github.com/mrirecon/myocardial-t1-mapping.

## Abbreviations

ANOVA: Analysis of variance
BART: Berkeley advanced reconstruction toolbox
bpm: Beats per minute
bSSFP: Balanced steady State Free Precession
CMR: Cardiovascular magnetic resonance
CPU: Central processing unit
CV: Coefficient of variation
FISTA: Fast Iterative Shrinkage Thresholding Algorithm
FLASH: Fast low-angle shot
FOV: Field of view
GPU: Graphics processing unit
IR: Inversion-recovery
IRGNM: Iteratively regularized Gauss-Newton method
MOLLI: Modified Look-Locker inversion recovery
NLINV: Nonlinear inversion
PCA: Principle component analysis
SAPPHIRE: Saturation pulse prepared heart-rate-independent inversion recovery
SASHA: SAturation recovery Single-sHot Acquisition
SD: Standard deviation
ShMOLLI: Shortened Modified Look-Locker inversion recovery
SNR: Signal-to-noise ratio
TE: Echo time
TR: Repetition time
XD-GRASP: EXtra Dimension-Golden angle Radial Sparse Parallel
