# Supplementary figures and images for: Model-based myocardial T1 mapping with sparsity constraints using single-shot inversion-recovery radial FLASH cardiovascular magnetic resonance
Source: J Cardiovasc Magn Reson. 2019 Sep 19;21:60. doi: 10.1186/s12968-019-0570-3 (PMC6751613; doi:10.1186/s12968-019-0570-3)

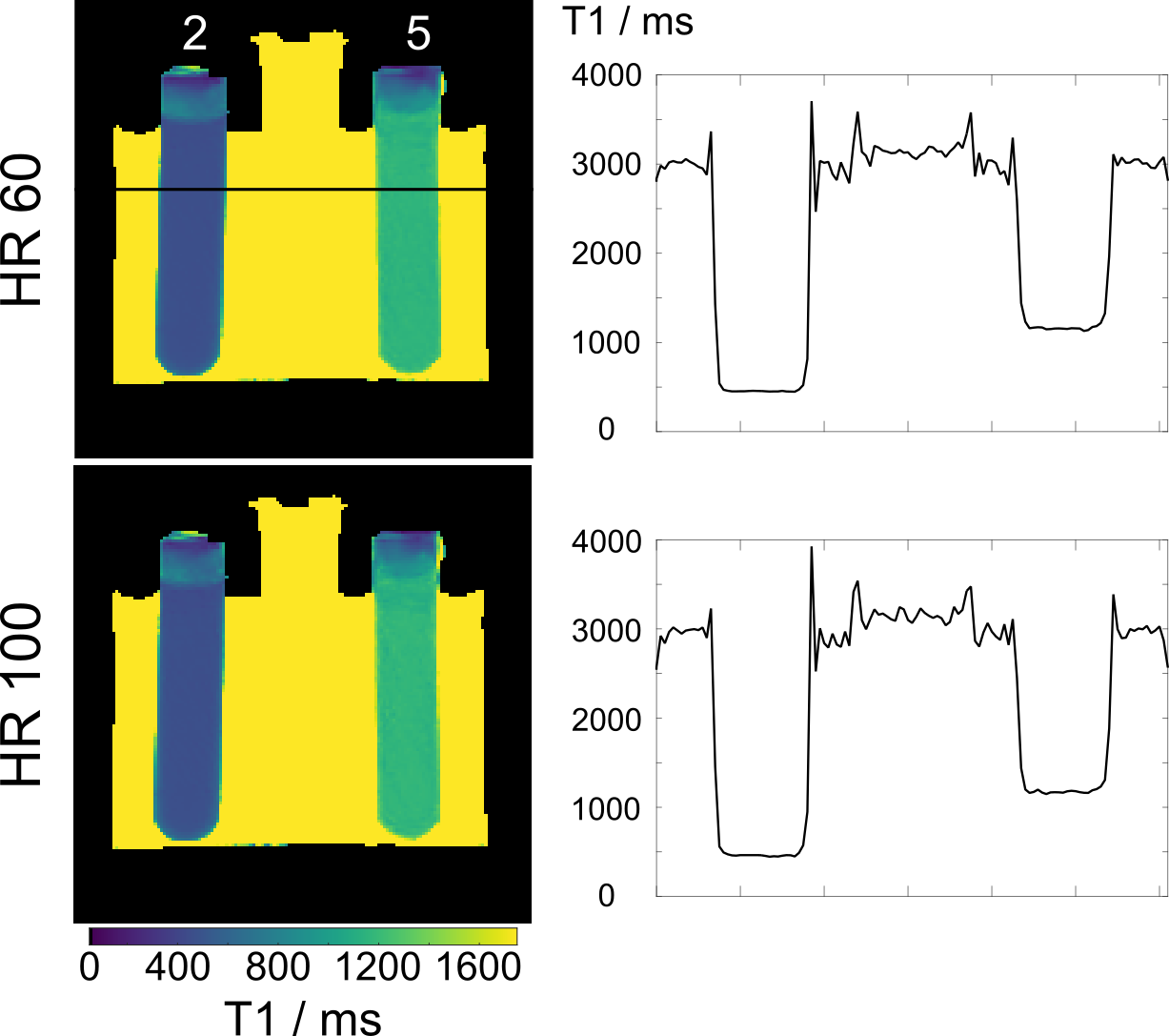

Supplement: Supplementary file 1 — Figure S1. Model-based long-axis T1 maps at heart rates (left top) 60 and (left bottom) 100 as well as (right) the corresponding T1 line profiles for the experimental phantom study. The quantitative T1 values are in the Additional file 3: Table S1. (PNG 100 kb) [file 12968_2019_570_MOESM1_ESM.png]

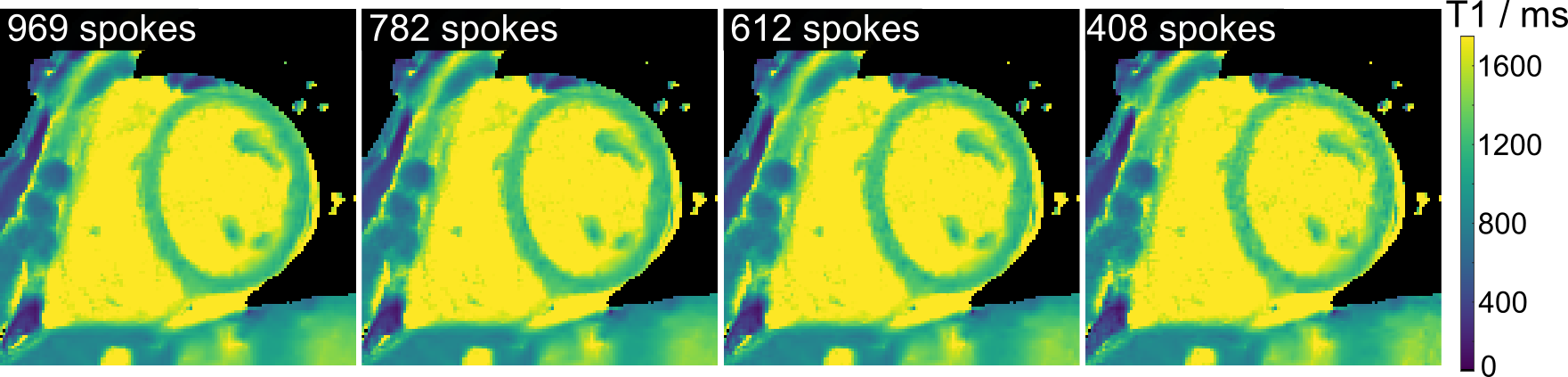

Supplement: Supplementary file 2 — Figure S2. Myocardial T1 maps on a healthy subject by retrospectively rejecting an increasing amount of data prior to model-based reconstructions. The amount of data deleted corresponds to heart rates 50, 60, 80, 100 bpm, respectively. The ROI-analyzed septum T1 values are 1251 ± 41 ms, 1235 ± 43 ms, 1236 ± 49 ms and 1274 ± 53 ms for each reconstruction. (PNG 149 kb) [file 12968_2019_570_MOESM2_ESM.png]
