# Supplementary material for: Model-based myocardial T1 mapping with sparsity constraints using single-shot inversion-recovery radial FLASH cardiovascular magnetic resonance
Source: J Cardiovasc Magn Reson. 2019 Sep 19;21:60. doi: 10.1186/s12968-019-0570-3 (PMC6751613; doi:10.1186/s12968-019-0570-3)
Supplement: Supplementary file 3 — Table S1. Long-axis T1 relaxation times (ms) for an experimental phantom and simulated heart rates 60 and 100. (DOCX 13 kb) [file 12968_2019_570_MOESM3_ESM.docx]

**Additional Table 1.** Long-axis T1 relaxation times (ms) for an experimental phantom and simulated heart rates 60 and 100.

| Tube | HR 60 | HR 100 |
| --- | --- | --- |
| 2 | 455 ± 4 | 457 ± 6 |
| 5 | 1155 ± 12 | 1161 ± 15 |
